# Supplementary material for: Stability of N-type inactivation and the coupling between N-type and C-type inactivation in the Aplysia Kv1 channel
Source: Pflugers Arch. 2024 Jul 15;476(10):1493–516. doi: 10.1007/s00424-024-02982-5 (PMC11639194; doi:10.1007/s00424-024-02982-5)
Supplement: Supplementary file 1 — (pdf 35 KB) [file 424_2024_2982_MOESM1_ESM.pdf]

Stability of N-type inactivation and the coupling between N-type and C-type inactivation in the *Aplysia* Kv1 channel

Tokunari Iwamuro, Kazuki Itohara and Yasuo Furukawa

Laboratory of Neurobiology, Graduate School of Integrated Sciences of Life,
Hiroshima University, Kagamiyama 1-7-1,Higashi-Hiroshima 739-8521, Japan.

running title: the coupling of N-type and C-type inactivation in *Aplysia* Kv1 channel

All the correspondence should be addressed:

Yasuo Furukawa, PhD
Laboratory of Neurobiology
Graduate School of Integrated Sciences of Life
Hiroshima University
Kagamiyama 1-7-1, Higashi-Hiroshima 739-8521, Japan
Tel: +81 82-424-6566
Fax: +81 82-424-0734
e-mail: yasfuru@hiroshima-u.ac.jp

Supplementary tables

Table 1
Table 2

Table 1. Cumulative inactivation assessed by exponential fittings

		AKv1				I8Q			
solution	ip	A1	$\tau1$	A2	$\tau2$	A1	$\tau1$	A2	$\tau2$
ND96	-40	0.36±0.10	8.9±1.7	-0.69±0.03	4201.6±639.8	0.19±0.05	6.1±0.9	-0.30±0.06	209.6±29.3
ND96	-80	0.14±0.03	3.9±1.2	-0.53±0.06	1476.9±87.3	-	-	-0.22±0.05	78.83±4.5
ND96	-120	-	-	-0.45±0.07	602.2±53.1	-	-	-0.19±0.04	40.9±0.8
ND96	-160	-	-	-0.41±0.08	262.6±33.1	-	-	-0.15±0.04	31.6±1.9
high K^+	-40	0.25±0.06	5.6±2.0	-0.57±0.04	1037.7±184.8	0.19±0.02	5.7±0.8	-0.26±0.04	72.4±13.1
high K^+	-80	-	-	-0.46±0.07	271.1±26.3	-	-	-0.19±0.03	29.7±2.2
high K^+	-120	-	-	-0.42±0.08	80.8±5.9	-	-	-0.18±0.03	12.8±1.3
high K^+	-160	-	-	-0.39±0.08	29.4±2.0	-	-	-0.17±0.03	5.4±0.4

Mean±SD of parameters estimated by exponential fitting of the relationship between I_{2nd}/I_{1st} and the inter-pulse interval are shown (n=5).

ip: inter-pulse potential (mV)

A1, A2: the relative amplitudes of exponential components

$\tau1$, $\tau2$: time constants (msec) of exponential components

Relative amplitude of maximum recovery (Y0) was assigned to 1

Table 2. Exponential recovery of the ON gating charge

channel	ip	A1	τ1	Y0	n
AKv1	-80	-0.463±0.057	31.4±5.4	0.572±0.060	9
	-100	-0.635±0.067	17.2±3.0	0.744±0.048	9
	-120	-0.754±0.084	8.5±1.8	0.838±0.055	9
	-140	-0.846±0.092	4.1±1.0	0.868±0.059	9
I8Q	-80	-0.820±0.118	31.2±2.6	0.905±0.060	4
	-100	-0.915±0.081	15.8±1.6	0.972±0.026	4
	-120	-0.982±0.092	7.9±1.5	1.011±0.041	4
	-140	-1.081±0.123	4.0±0.8	1.024±0.057	4
ΔN	-80	-0.973±0.029	32.1±1.1	1.005±0.028	3
	-100	-0.999±0.031	15.3±0.8	1.014±0.031	3
	-120	-0.987±0.035	6.8±0.5	0.994±0.034	3
	-140	-0.998±0.032	3.5±0.6	1.002±0.032	3

Mean±SD of parameters estimated by a single exponential fitting (see Materials and methods) of the relationship between the relative amplitude of the gating charge at 2nd pulse (Q2/Q1) and the inter-pulse interval are shown.

ip: inter-pulse potential (mV)

A1: relative amplitude of an exponential component

τ1: time constant (msec)

Y0: relative amplitude of an estimated maximum recovery

n: the number of tested oocytes
